# Introducing the Adsorption Energy Distribution Calculation for Two-Component Competitive Adsorption Isotherm Data

**DOI:** 10.1021/acs.analchem.4c04663

**Published:** 2025-01-21

**Authors:** Abdul Haseeb, Yosief Wondmagegne, Miguel X. Fernandes, Jörgen Samuelsson

**Affiliations:** † Department of Engineering and Chemical Sciences, Karlstad University, SE-651 88 Karlstad, Sweden; ‡ Department of Mathematics and Computer Science, Karlstad University, SE-651 88 Karlstad, Sweden

## Abstract

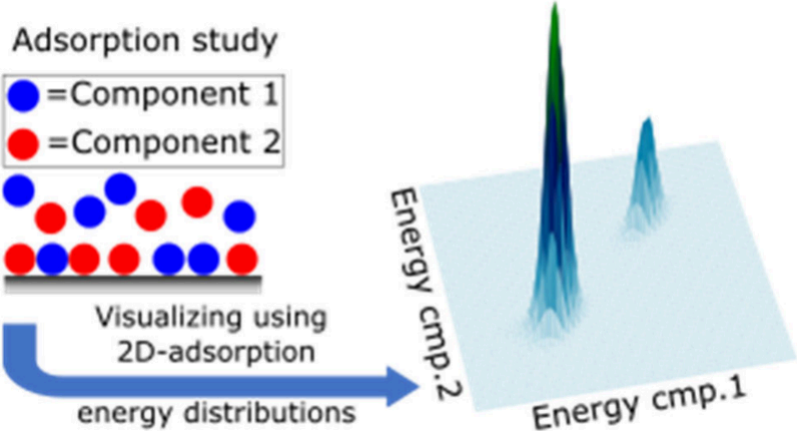

This work introduces the Adsorption Energy Distribution (AED) calculation using competitive adsorption isotherm data, enabling investigation of the simultaneous AED of two components for the first time. The AED provides crucial insights by visualizing competitive adsorption processes, offering an alternative adsorption isotherm model without prior assuming adsorption heterogeneity, and assisting in model selection for more accurate retention mechanistic modeling. The competitive AED enhances our understanding of multicomponent interactions on stationary phases, which is crucial for understanding how analytes compete on the stationary phase surface and for selecting adsorption models for numerical optimization of preparative chromatography. Here, the two-component AED was tested on both synthetic and experimental data, and a very successful outcome was achieved.

## Introduction

In liquid chromatography, analytes interact in many different ways with the stationary phase surface, each interaction having a different strength (adsorption energy). This phenomenon gives rise to adsorption heterogeneity. In chromatography, it is important to note that heterogeneity is not just a property of the stationary phase surface; it is a property of the whole phase system, i.e., the analyte, the mobile phase and the stationary phase.^1^ In analytical chromatography, this adsorption heterogeneity could result in peak tailing, reduced resolution, excessively long retention times for basic and cationic compounds, unexpected retention shift, and decreased sensitivity.^2^ Heterogeneity in the stationary phase can arise from impurities (e.g., boron, iron) accumulating on the surface, unsatisfied and strained valences of surface atoms in the silica matrix;^1^ residual silanols, variation in the C18-bonded layer, and large cavities within this layer.^3^

The retention mechanism in analytical chromatography can be understood through adsorption isotherms, which are crucial for elucidating the different interactions between the analyte and the stationary phase that govern retention. Adsorption isotherms describe the relationship between the amount of analyte adsorbed onto the stationary phase and its concentration in the mobile phase under specific and constant temperature. An adsorption isotherm obtained under operating conditions in a chromatographic system can offer more realistic insights into the adsorption process than material science methods such as spectroscopic techniques, including NMR. However, some difficulties are associated with this approach. First, adsorption data can be described well by many rival adsorption isotherm models, and it becomes statistically difficult to determine which model is most suitable. Second, a selected adsorption isotherm may have a very good to excellent statistical fit to the experimental adsorption isotherm data but it may not be physically meaningful.^4^

Adsorption Energy Distribution (AED) is an extension of an adsorption isotherm to a continuous distribution of independent homogeneous adsorption sites across the range of adsorption energies under investigation. AED gives a better picture of the adsorption process by visualizing the number of adsorption sites and their associated adsorption energies. AED helps in selecting the most suitable adsorption model among the many equivalent models. For example, the adsorption isotherm of phenol and caffeine in methanol/water as a mobile phase on the Kromasil C18 column can be described by either Tóth or the Bi-Langmuir isotherm model. Statistical analysis suggests that the Tóth model fits best to the data. However, the AED calculated from raw adsorption data suggests a bimodal energy distribution, a result which is not compatible with Tóth model and points out that Bi-Langmuir is the better model.^4^

AEDs for single components, hereafter referred to as one-comp AED, have been estimated for various adsorption processes such as Langmuir, Bi-, Tri-, and Quad-Langmuir, Jovanovic, Tóth, and others, for a variety of analytes.^1,3^ A similar mathematical approach has been utilized for kinetic study,^5^ but to the best of our knowledge, AED for competitive adsorption processes has not been calculated and reported until now. we know that chromatographic separation is always a multicomponent phenomenon in which two or more components interact and compete with each other for the stationary phase surface. Consequently, these components will affect each other’s adsorption, which may have profound effects on the overall separation process. Therefore, determining a competitive AED would provide additional insight into the separation process. Just as one-comp AED aids in selecting accurate isotherm models and visualizing adsorption heterogeneity for a single component, two-component AED (two-comp AED) will become a valuable tool for selecting the appropriate competitive isotherm model and visualizing adsorption heterogeneity for two components simultaneously.

The aim of this study is to calculate the AED for competitive adsorption processes. For the first time, we develop an algorithm to calculate and visualize the AED for a two-component adsorption process by introducing two-comp AED. Using syntenic data, two-comp AED is calculated for adsorption isotherm data describing adsorption processes with varied numbers of adsorption sites (Langmuir, Bi-Langmuir, and Tri-Langmuir). We also discuss the importance of the concentration interval in adsorption isotherm acquisition. Finally, the two-comp AED is calculated for several experimental adsorption isotherm data sets to visualize and analyze the competitive adsorption processes. It is important to note that the aim of this study is not to conduct an in-depth investigation of two-comp AED, but rather to introduce the concept and demonstrate its usefulness for visualizing competitive adsorption processes.

## Theory

A commonly used adsorption isotherm model is the Langmuir model.^6^ It assumes ideal adsorption sites with just one adsorption energy. However, adsorption often deviates from this ideal behavior, resulting in a heterogeneous adsorption energy. This heterogeneity may appear as multiple independent sites with different adsorption energies or as a unimodal heterogeneous energy distribution. The *n*-Langmuir model (eq 1), which represents multiple independent ideal adsorption sites, extends the Langmuir model by describing each of the *n* sites individually.
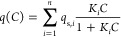
1where *q* is the analyte concentration adsorbed on the stationary phase, *C* is the analyte concentration in the mobile phase, *n* is the number of independent adsorption sites, *q*_s,*i*_ is monolayer saturation capacity of the *i*^th^ site, and *K*_*i*_ is the association equilibrium constant for the *i*^th^ site. When *n* = 1, we obtain the Langmuir model; for *n* = 2, the Bi-Langmuir model; for *n* = 3 the Tri-Langmuir model, and so on.

**Figure 1:**
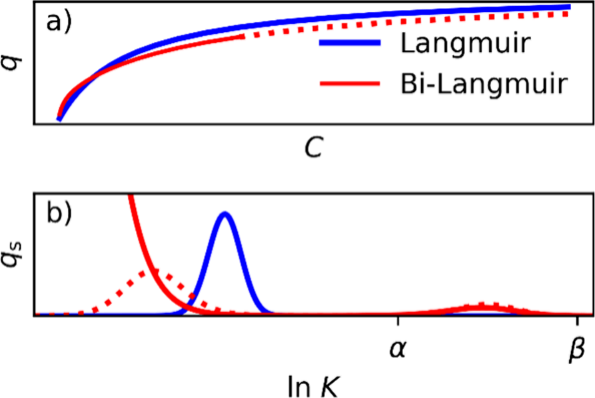
Schematic of (a) Langmuir and Bi-Langmuir adsorption isotherms (b) with corresponding one-comp AED. The solid red line excludes high concentration data, whereas the dotted red line includes the full data set. α and β are integration limits; see eq 4.

In Figure 1a, a typical Langmuir adsorption isotherm (blue line) and a Bi-Langmuir adsorption isotherm are plotted (red line). The dotted red line shows the Bi-Langmuir over a broader concentration range and the solid red line represents a smaller concentration interval. From a separation perspective, analytes described by the Langmuir model seldom exhibit peak tailing. On the other hand, analytes described by the Bi-Langmuir model often show peak tailing.^7^

One valuable tool for visualizing the adsorption heterogeneity is to plot the AED. Essentially, AED provides a framework for understanding adsorption as a spectrum of adsorption energies, offering a more comprehensive view of the adsorption process. In general, AED is a continuous distribution of independent homogeneous sites across the range of adsorption energies (*f*(ln *K*)), which determines the total amount of analyte adsorbed through the integral over the energy space, *D*.
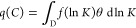
2

*D*-space is often spanned from ln *K*_min_ to ln *K*_max_, where *K*_min_ = 1/*C*_max_ and *K*_max_ = 1/*C*_min_. Here *C*_min_ and *C*_max_ represent the lowest nonzero concentration and the highest concentration in the experimental adsorption data. Various kernel functions (θ) can be used to obtain a solution for eq 2. The Langmuir kernel function (eq 3) is most commonly used; however, it works only for type I adsorption isotherms,^8^ which are convex and do not contain any inflection points; see Figure 1a for example.^9^
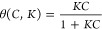
3

In other words, eq 2 using the Langmuir kernel function (eq 3) is similar to an *n*-Langmuir model (eq 1) with an infinite number of independent adsorption sites. The goal is to find the function *f*, which describes the monolayer saturation capacity for each of these sites. Equation 2, in this way, provides an alternative adsorption isotherm model without the need to assume adsorption heterogeneity.

The AED for a Langmuir model is a unimodal symmetrical distribution (Figure 1b, blue line), with the distribution area corresponding to the monolayer saturation capacity, and has an apex at the energy linked to the equilibrium constant, ln *K*. In contrast, the Bi-Langmuir model shows a bimodal distribution (Figure 1b dotted red line). Each distribution corresponds to a specific adsorption energy and has an area corresponding to the site’s monolayer saturation capacity The natural logarithm of the equilibrium constant, *K*, is utilized in the integral (eq 2) because the adsorption energy exhibits an exponential relationship with the equilibrium constant, as per the Arrhenius equation.

To determine the saturation capacity of a specific site, one must integrate over its specific AED:
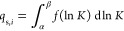
4where α and β are integration limits that cover the particular adsorption site in question. This is depicted in Figure 1b, for the high energy site in the bimodal distribution.

If *C*_max_ is not high enough to span the energy space used in the AED calculation to cover the weak interactions, weak adsorption sites will not be resolved; see Figure 1b (solid red line) for our Bi-Langmuir case. However, the high energy site is resolved because it lies within the energy space considered in the AED calculation. When *C*_max_ is sufficient to span the energy space, all sites will be resolved, as seen in Figure 1b (red dotted line); see the Supporting Information for more details.

During chromatographic separation, several compounds compete for the available surface space on the adsorbent. To describe this process, competitive adsorption isotherms are used. A commonly used model is the competitive Langmuir model, and its expression for two components is as follows:
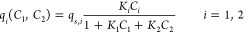
5where (*q*_*i*_) is the adsorbed amount of the *i*th compound and *C*_1_ and *C*_2_ are the concentrations of compounds 1 and 2 in the mobile phase. The index of the equilibrium constant represents the equilibrium constant for compounds 1 and 2.

The integral version of the adsorption isotherm in eq 2 covers only one energy space associated with *K*. In contrast, eq 6 extends this to a two-component adsorption isotherm. Additionally, the independent homogeneous sites used in the two-comp AED calculations contain two distinct adsorption energy spaces, corresponding to *K*_1_ and *K*_2_.
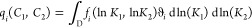
6*D* denotes a bound region in the plane over which the integral is calculated. Similar to eq 2, this is now applied to each energy dimension.

Note that we will have one AED (*f*_*i*_) for each component under investigation, two in our case. In this study, the two-component Langmuir model *ϑ*_*i*_ is used as a kernel function:
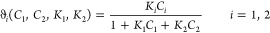
7This kernel function is limited to studying only Type I competitive adsorption isotherms. Notably, since the kernel function includes two distinct energy parameters (*K*_1_, *K*_2_), it inherently results in eq 6 being a double integral, regardless of the number of adsorption sites present in the solution of eq 6.

Equations 2 and 6 lack analytical solutions, and numerical solutions are required. In this study the maximum-likelihood method, called expectation-maximization (EM), is used to solve the one-comp AED presented in Figure 1b, in an iterative way.^6,10^ Similarly, an algorithm for solving the two-comp AED was developed. Instead of a double integral (eq 6), the EM will be a double sum over ln *K*_1_ and ln *K*_2_ adsorption energy spaces. In the calculation, the energy space is divided into a uniform grid (using a number of grid points in each energy dimension); see the Supporting Information for more details about the numerical solutions, including discussion about convergence.

## Experimental Section

**Figure 2:**
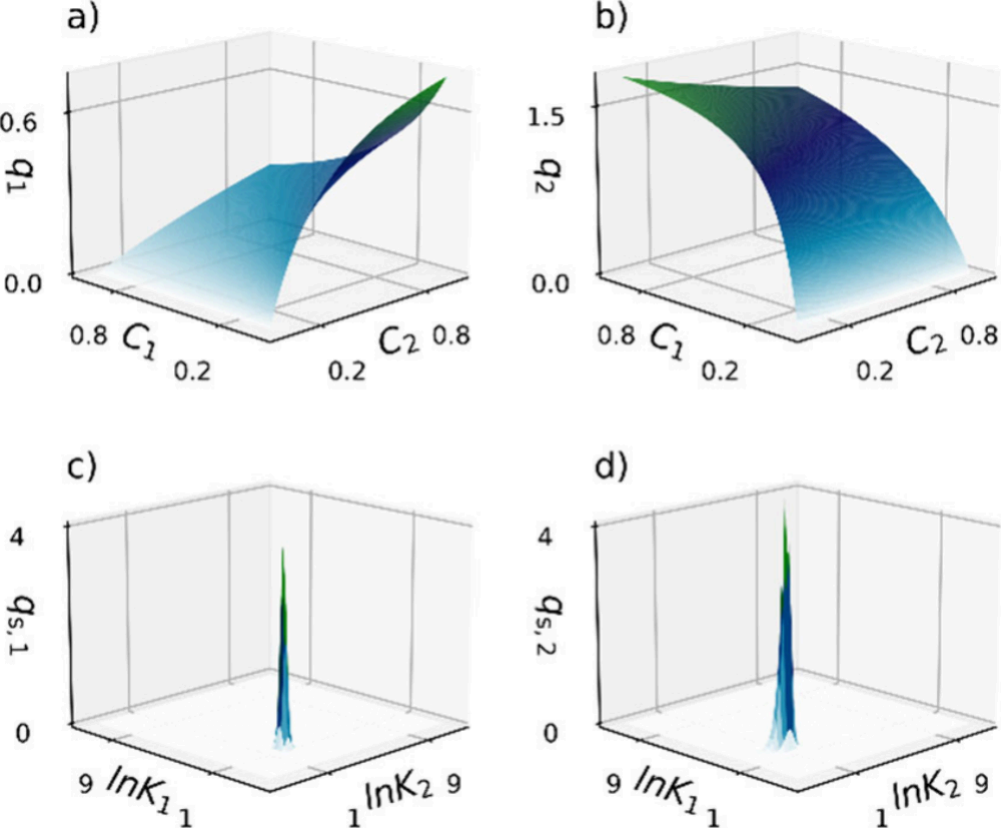
Two-component Langmuir adsorption isotherms (a, b) and their corresponding two-comp AED (c, d). (a and c) for component 1 and (b and d) for component 2 two-comp AED calculations using 100,000 iterations.

The mobile phase concentration data used for adsorption isotherms are presented in Figure S2 for the synthetic data. For experimental systems 1 and 2, the mobile phase concentrations are shown in Figures S3 and S4 respectively, in the Supporting Information. Adsorbed concentrations for the synthetic data were calculated using Langmuir, Bi-Langmuir, and Tri-Langmuir models. Saturation capacity was set to 1 for each site, except for the second compound in Figure 2 where it was set to 2.

The two-comp AED was calculated using the EM method. The algorithm was implemented by using Python 3.10 with the numpy, numba, and matplotlib libraries for visualization. With 100 grid points in each energy space, the specific number of iterations used in the calculation is presented in the Results and Discussion. The nonlinear least-squares model fittings were performed using the Levenberg–Marquardt algorithm, implemented in the Python lmfit library. Areas were calculated using the trapezoidal rule, implemented in the numpy library.

The adsorption isotherm parameters from the two-comp AED were estimated by locating the *K*-values at the apex of the distributions and determining the monolayer saturation capacity by integrating them over the AED distribution.

## Results and Discussion

### Synthetic Data

In this section, the two-comp AED is validated using synthetic data with known adsorption isotherms.

In Figure 1, we present the single-component adsorption isotherms for the Langmuir and Bi-Langmuir models, along with their corresponding one-comp AEDs. Figure 2 shows the two-component competitive Langmuir adsorption isotherms (panels a and b) and their corresponding two-comp AEDs ( panels c and d). The model parameters used here are ln *K*_1_ = 1, ln *K*_2_ = 2, *q*_s,1_ = 1, and *q*_s,2_ = 2.

For the two-component case, we will have an adsorption isotherm describing the adsorption competition and a two-comp AEDs, for each component. Observe that a two-comp AED is a 3D plot, where the energy axes ln *K*_1_ and ln *K*_2_ span the energy space (*D* in eq 6), and the *q*_s_-axis represents the abundance of each adsorption site. From the adsorption isotherms alone, it is difficult to reveal adsorption heterogeneity. However, the two-comp AEDs clearly show that both components exhibit a single symmetrical distribution, indicating unimodal and homogeneous adsorption. Additionally, the AED volume for the second component (Figure 2d) is much larger than that of the first component (Figure 2c). This suggests a higher monolayer saturation capacity for the second component, which matches the set values used in the two-component AED calculation.

A feature of the two-component AED is that it requires adsorption isotherms determined from binary-component solutions for conversion. It does not rely on single-component adsorption isotherm data, and using only single-component data results in an unconverged AED, as illustrated in Figure S1 in the Supporting Information.

**Figure 3:**
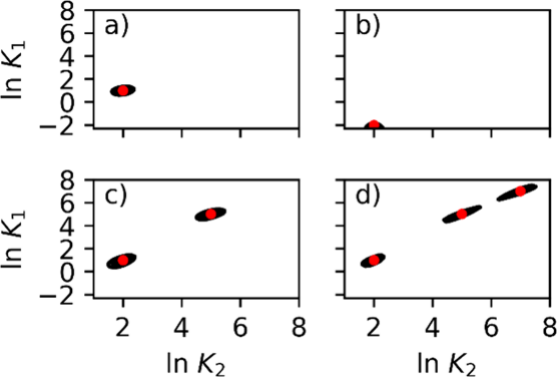
Calculated two-comp AED for the first component using competitive *n*-Langmuir models with (a) ln *K*_1_ = 1 and ln *K*_2_ = 2, (b) ln *K*_1_ = −2 and ln *K*_2_ = 2, (c) two sites the first with ln *K*_1_ = 1 and ln *K*_2_ = 2 and the second ln *K*_1_ = 5 and ln *K*_2_ = 5, and (d) three sites, the first with the first with ln *K*_1_ = 1 and ln *K*_2_ = 2, the second ln *K*_1_ = 5 and ln *K*_2_ = 5, and the third with ln *K*_1_ = 7 and ln *K*_2_ = 7. Red dots indicate the set model *K*-values. Iterations used in the calculations were 100,000 for (a)–(c) and 1,000,000 for (d).

Another way to visualize the two-comp AED is with contour plots, which are projections from above the 3D plots shown in Figure 2. Figure 3a illustrates this projection from Figure 2c, with the red dots indicating the set association equilibrium constants. Figure 1 highlights the importance of acquiring adsorption isotherm data at sufficiently high concentrations to resolve the low-energy sites. To investigate this, one could either lower the maximum concentrations used in the adsorption isotherm acquisition (*C*_max_) or reduce the equilibrium constant, thereby requiring a higher *C*_max_. In Figure 3b, the equilibrium constant for the first compound (*K*_1_) was reduced, needing higher concentrations to resolve that adsorption site (see Figure 3b). As shown in the figure, the two-comp AEDs are located near the lower ln *K*_1_ boundary and fail to converge into a proper distribution.

The strength of one-comp AED calculations is their ability to visualize the number of adsorption sites. To test whether this is also the case for two-comp AED, competitive Bi-Langmuir and Tri-Langmuir adsorption data were generated, and the two-comp AED values were calculated, as shown in Figure 3c,d, respectively. The Tri-Langmuir case required more iterations (1,000,000) than the Langmuir and Bi-Langmuir cases (100,000) to achieve good convergence. In both cases, the association equilibrium constants and the monolayer saturation capacities were predicted accurately.

Increasing the number of iterations in the AED calculation produces more compact distributions, allowing closely situated energy sites to be resolved. However, it does not affect the predicted saturation capacity or the association equilibrium constants.

### Two-Comp AED Calculations Based on Experimental Data

In experimental settings, noise is inevitable, owing to the random errors in the measurements. To test the usefulness of the two-comp AED for experimental data, the competitive adsorption data from two different experimental data sets here called systems 1 and 2 were investigated.

System 1: Tracer pulse two-component adsorption isotherm data for methyl mandelate (MeM) and ethyl mandelate (EtM) on an Eclipse XDB-C8 column were taken from Samuelsson et al.^11^ The eluent used was 30/70 (v/v) acetonitrile/water. Each adsorption isotherm consists of 36 data points.

**Figure 4:**
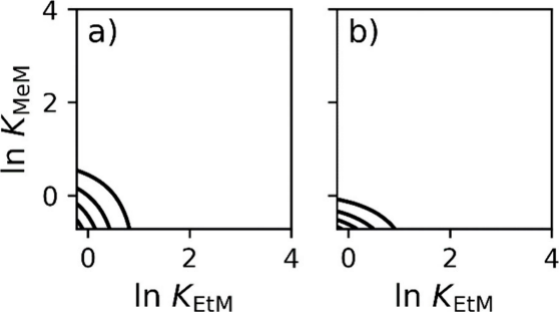
Calculated two-comp AED for adsorption data for system 1. The two-comp AED for (a) MeM and (b) EtM. Calculations used 10,000 iterations.

In Figure 4a, the two-comp AED for MeM is presented, and in Figure 4b, the two-comp AED for EtM is shown. In this case, the AEDs do not converge to a distribution but are located in the low-energy space. This is because the concentration used in the adsorption isotherm acquisition was not sufficiently high to resolve this interaction, which is comparable to that in Figure 3b. The *K*_min_ for MeM was 4.88 M^–1^ and for EtM was 8.00 M^–1^ for the concentration ranges used in the given experiment. By integrating over the two-comp AED distribution, the monolayer saturation capacities were determined to be 2.33 and 2.23 M for MeM and EtM, respectively. These results are close to the reported adsorption isotherm parameters, indicating that the two-comp AED provides accurate results. The results of the two-comp AED and model fit are presented in the Supporting Information (Table S1).

System 2: Frontal analysis (FA) and frontal analysis by elution by characteristic points (FACP) two-component adsorption isotherm data of benzyl alcohol (BA), 2-phenylethanol (PE), and 2-methylbenzyl alcohol (MBA) on a Symmetry C18 column were taken from Quiñones et al.^12^ The eluent used was 50/50 (v/v) methanol/water. Each adsorption isotherm contained 130 data points for FACP and 50 data points for FA.

**Figure 5:**
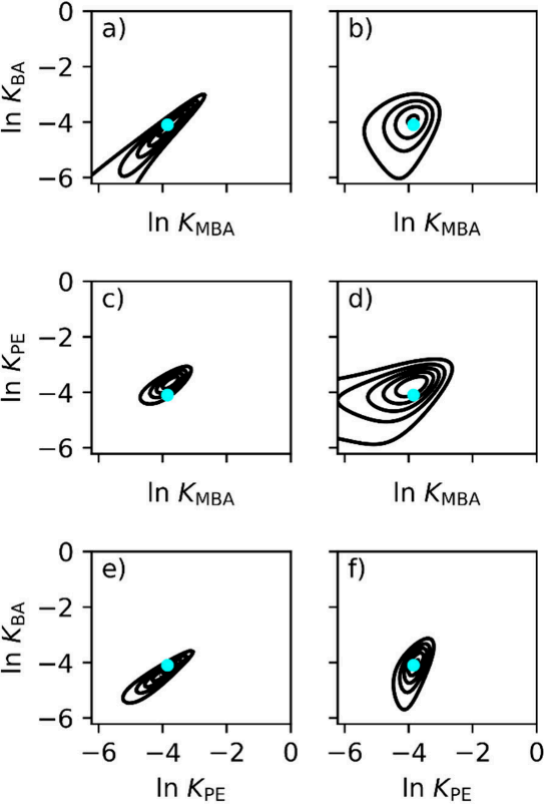
Calculated two-comp AED from adsorption data for system 2. The two-comp AED for (a) BA and (b) MBA for the BA/MBA system, (c) PE and (d) MBA for the PE/MBA system, and (e) BA and (f) PE for the BA/PE system. Calculations used 100,000 iterations The cyan dots indicate the estimated *K* values from the model fit to the competitive Langmuir model.

Owing to the complexity of data set in system 2, the number of iterations was increased to 100,000. For the PE/BA system, the adsorption site did not converge with FACP data; therefore, we used the FA data set instead, which resolved the issue The resulting two-comp AED is presented in Figure 5, where the cyan dots in the figures are estimated equilibrium constants from the model fit using the competitive Langmuir model (eq 7) to the experimental data; see Table S1 for parameters as well as corresponding estimated two-comp AED parameters.

All two-comp AEDs in Figure 5 are unimodal, suggesting that a unimodal adsorption isotherm model should be used to describe the adsorption process. However, when comparing the two-comp AEDs in Figure 5 with those from the synthetic data in Figure 3a, all components show similar distributions except for MBA, which exhibits a broader asymmetric distribution. This indicates that the simple competitive Langmuir model is insufficient for describing the adsorption process.

When the estimated *K* and *q*_s_ values from the model fit using nonlinear regression are compared with those from the two-comp AED calculations, a considerable difference is observed. The *q*_s_ values differ by an average of 24%, whereas the *K* values show an 8.5% difference. This also indicates that the simple competitive Langmuir model is insufficient for describing the adsorption process. This aligns well with the findings of Quiones et al.^12^ In their study, they developed an adsorption isotherm based on nonideal adsorbed solution theory to fit the same experimental data, noting that their model reduced to a Langmuir-type expression only under certain conditions. However, their model development was primarily driven by statistical improvements in their model fit. With the two-comp AED, we can now visualize the complex adsorption process, providing a much clearer understanding of the adsorption dynamics.

It is important to note that when two separate single-component adsorption isotherms (one for each component) fully capture the competition, the two-comp AED will not provide any new insights. This is the case in Figure 3 (where the conditions are set) and possibly in Figures 4 and 5a,c,e,f. However, for more complex adsorption scenarios, as illustrated in Figure 5b,d, the two-comp AED offers a clear advantage. In all experimental cases, it eliminates the need for prior knowledge of competitive interactions, as this information is revealed through the tool itself.

## Conclusions

The two-comp AED provides several benefits. First, it visualizes competitive adsorption processes, which can be crucial for understanding separation processes. Many chiral separations, described by the type I model, could be currently studied using the two-comp AED. Second, it serves as an alternative adsorption isotherm model. Third, the tool aids in selecting the appropriate adsorption isotherm model for fitting raw data, simplifying the model selection. Fourth, it helps determine whether the chosen concentration interval is sufficient for reliable adsorption isotherm modeling.

The two-comp AED shares the drawbacks of the one-comp AED, including longer computation times. It also requires accurate two-component adsorption isotherm data, which can be difficult to obtain.

Finally, as this is the first study to explore this method, the convergence criteria and required raw data are not yet fully understood.

## Supporting Information Available

Algorithm for calculating the one-comp and two-comp AED, convergence criteria discussion, raw concentration data used in calculations and model fits (PDF)
