# Supplementary material for: Introducing the Adsorption Energy Distribution Calculation for Two-Component Competitive Adsorption Isotherm Data
Source: Anal Chem. 2025 Jan 21;97(4):1966–71. doi: 10.1021/acs.analchem.4c04663 (PMC11800172; doi:10.1021/acs.analchem.4c04663)
Supplement: Supplementary file 1 — ac4c04663_si_001.pdf [file ac4c04663_si_001.pdf]

Supporting information for:

Introducing the Adsorption Energy Distribution Calculation for Two-Component Competitive Adsorption Isotherm Data

Abdul Haseeb[1], Yosief Wondmagegne[2], Miguel X. Fernandes[1], Jörgen Samuelsson[1,]*

[1.] Department of Engineering and Chemical Sciences, Karlstad University, SE-651 88 Karlstad, Sweden.
[2.] Department of Mathematics and Computer Science, Karlstad University, SE-651 88 Karlstad, Sweden.

Table of Contents

Numerical calculation of adsorption energy distribution 1
One-component AED numerical scheme 1
Two-component AED numerical scheme 2
Remarks regarding the convergence criteria, two-component case 3
Concentration data used in all calculations 5
Model fits 7
Adsorption isotherm model fit using the two-component AED 7
References 9

Numerical calculation of adsorption energy distribution

This section provides a detailed explanation of how the Adsorption Energy Distribution (AED) is calculated. We present a quick recap of the updating stage in a classical scheme for one-component adsorption isotherm data, followed by the new two-component AED approach for competitive two-component adsorption isotherm data. In the end a section covering convergence and demand on the experimental data is presented.

One-component AED numerical scheme

In integral form a general expression of an heterogeneous adsorption isotherm can be formulated as:

$$q(C) = \int_{\mathrm{D}} f(\ln K) \cdot \theta(C\,, K)\, d\ln K \qquad \text{(S1)}$$

where $q(C)$ is the adsorption isotherm, f is the adsorption energy distribution (this is the target of the calculation), and $\theta(C, K)$ the kernel function, and in this study the Langmuir kernel was used, and it can be expressed as:

$$\theta(C,K) = \frac{KC}{1 + KC} \tag{S2}$$

K is the association equilibrium constant and C is the concentration of the solute in the mobile phase.

The adsorption integral equation, Eq S1, which describes the relationship between the measured adsorption isotherm q and the unknown adsorption energy distribution function f, lacks a solution in a closed form which needs numerical approximation. This is achieved by rewriting the integral (eq S1) to a sum. Several algorithms have been proposed for this purpose and one that has gained ground is the maximum-likelihood method, called expectation-maximization (EM), because it is rather stable. [1,2] The updated expression for a particular energy ($\ln K_i$) is presented in eq S3.

$$f^m(ln\, K_i) = f^{m-1}(ln\, K_i) \sum_{j=1}^{n} \frac{q_{\text{exp}}(C_j)}{q_{\text{calc}}(C_j)} \theta(C_j, K_i) \Delta\, ln\, K \tag{S3}$$

f^m is the new energy distribution and, f^{m-1} is the previous AED estimate, m is the iteration step, q_{exp} is the experimental adsorption isotherm, q_{calc} is the calculated adsorption isotherm data and is updated in each iteration step with a sum corresponding to eq S1. The limits or the energy spacing within which the AED is calculated are dictated by the concentrations in the raw adsorption isotherm data. Classically, one starts for $\ln K_{\text{min}}$ and sum up to $\ln K_{\text{max}}$ which are usually estimated by $K_{\text{min}} = 0.1/C_{\text{max}}$ and $K_{\text{max}} = 10/C_{\text{min}}$, respectively, where C_{min} and C_{max} are the lowest non zero concentration and the highest concentration used in the experimental adsorption isotherm data. [3,4] The energy space is divided into a uniform grid (using a number of grid points) where the difference between two consecutive grid points is $\Delta\, ln\, K$. The initial energy distribution is a uniform distribution across the energy space under consideration. The iteration is performed until the sum of the root mean square error between q_{calc} and q_{exp} is within a specified tolerance.

Two-component AED numerical scheme

The one-component AED described above only covers the energy space associated with a single component. However, since we have an equilibrium constant for each component, we must integrate over both energy spaces.

$$q_i(C_1, C_2) = \int_{\mathrm{D}} f_i(ln\, K_1, ln\; K_2)\, \vartheta_i(C_1, C_2, K_1, K_2)\, d\ln(K_1)\, d\ln(K_2)\;, \quad i = 1, 2 \tag{S4}$$

D denotes a bounded region in the plane and is related to the appropriate minimum and maximum possible energy values. The two-component competitive Langmuir model is used as kernel function ϑ_i (C_1, C_2, K_1, K_2), and it can be expressed as:

$$\vartheta_i(C_1, C_2, K_1, K_2) = \frac{K_i C_i}{1 + K_1 C_1 + K_2 C_2}\;, \quad i = 1, 2. \tag{S5}$$

In contrast to the case of the one-component AED, the two-component AED updated expression using the EM method will be a double sum over $\ln K_1$ and $\ln K_2$ adsorption energy spaces. The EM algorithm

used here was inspired by the work of Waluga *et al.*, where they analysed cofactor-coupled enzymatic two-substrate kinetics.[5] For a particular pair of energy values (ln $K_{1,j}$, ln $K_{2,l}$) this is here expressed as:

$$f^{m}(ln\,K_{1,\,i}\,,ln\,K_{2,\,j}) = f^{m-1}(ln\,K_{1,\,j}, ln\,K_{2,\,l}) \sum_{r=0}^{R} \sum_{p=1}^{P} \frac{q_{\mathrm{exp}}(C_{1,r},\,C_{2,p})}{q_{\mathrm{calc}}(C_{1,r},\,C_{2,p})} \vartheta_{i}(C_{1,r},\,C_{2,p},\,K_{1,\,j},K_{2,\,l})\,\Delta\,ln\,K_{1}\Delta\,ln\,K_{2} \tag{S6}$$

The integration limits in the energy space in which the two-component AED is calculated are dictated by the concentrations in the raw adsorption isotherm data, in the same manner as for the one-component AED. The energy space is also divided into a uniform grid (using a number of grid points in each energy dimension) where the difference between two consecutive grid points is $\Delta\,ln\,K_1$ in K_1 energy space and $\Delta\,ln\,K_2$ in K_2 space. The initial energy distribution is a uniform distribution across the energy spaces under consideration.

Remarks regarding the convergence criteria, two-component case

In a broader sense, both eq S2 and eq S4 belong to a class of Fredholm integral equations of the first kind which often results in ill-posed problems, that may have no solution, or if a solution exists it is not unique and may not depend continuously on the available data, namely q respectively q_i. Approximate solutions to such equations could be achieved using many different approaches such as regularization or as here the EM method.[2,6] The EM method, has shown to be an robust method because of its iterative nature and apply well to AED estimation from noisy data.[6]

A solution is obtained by iterating until the root mean square error between calculated and experimental q (adsorption isotherm) is within the experimental noise level, which is difficult to determine, until the distribution shows minimal change (a specific tolerance), or to a specific number of iterations, the latter two are more practical approaches. Excessive iterations can introduce noise across the AED and may lead to misleading conclusions from the calculations.[3]

Selecting the kernel function is also important so that it can handle the experimental adsorption isotherm data type. The Langmuir kernel used in this study can only be used to study type I adsorption isotherms. If different types of adsorption models are under investigation, one needs to select a different kernel function.

The energy space, D, for the Langmuir kernel function that are well characterized in one-component AED calculations, are often spaned between ln K_{min} to ln K_{max}, where $K_{\mathrm{min}} = 1/C_{\mathrm{max}}$ and $K_{\mathrm{max}} = 1/C_{\mathrm{min}}$, respectively.[4] The energy space is expanded several times to $K_{\mathrm{min}} = 0.1/C_{\mathrm{max}}$ and $K_{\mathrm{max}} = 10/C_{\mathrm{min}}$, to get better convergence, if the adsorption energy is close to the integration boundaries. Sometimes, the adsorption energy for a particular adsorption process is outside the used energy space. In such cases, this will result in unresolved distributions as illustrated in Figures 1b, 3b, and 4. This concentration requirement for the acquired adsorption isotherm is not unique to AED calculations but also applies to traditional non-linear regression model fitting for an adsorption isotherm model. Accurately estimating the equilibrium constant is challenging if the data is acquired outside the required concentration regions. Therefore, adsorption isotherm data should be collected over a broad concentration range to capture both weak interactions at high concentrations and strong interactions at low concentrations. Addressing this issue can only be solved by additional experiments, which can be impractical or even impossible in some cases due, for instance, to the limited analyte solubility in the mobile phase.

Regardless of the selected kernel function, the two-component AED will depend on the concentrations of both components. If the solution space is spanned using only single-component data and not binary-analyte solutions, much of the energy space will remain unexplored during the calculations. To investigate the need of competitive adsorption data, three different data sets were constructed: Data Set I includes both binary-component adsorption isotherm data as well as single-component adsorption isotherm data for each component separately (Figure S1a); Data Set II includes only binary-component adsorption isotherm data (Figure S1b); and Data Set III includes only single-component data for both components (Figure S1c). The corresponding two-component AEDs for component 1 are presented in Figures S1d, S1e, and S1f, respectively.

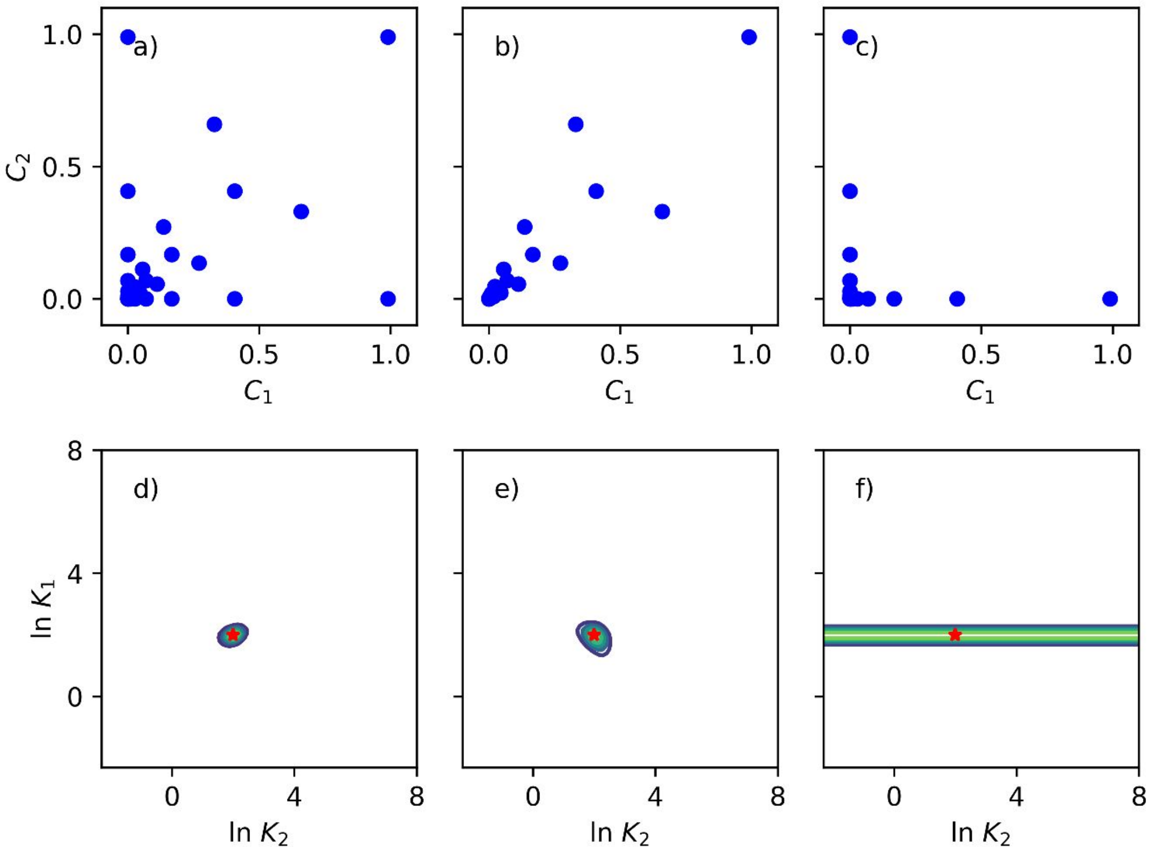

Figure S1: Mobile phase concentration data sets and corresponding two-component AEDs. (a–c) Concentration data for compounds 1 and 2: (a) Data Set I includes single-component isotherms and binary-component solutions of component 1 and 2, (b) Data Set II includes only binary-component adsorption isotherm data of component 1 and 2, and (c) Data Set III includes only single-component isotherms for component 1 and 2. (d–f) Two-component AEDs calculated for component 1 from (d) Data Set I, (e) Data Set II, and (f) Data Set III. The red star makes the set *K*-values for the adsorption isotherm Calculations were performed using 100,000 iterations, with the energy space spanned by 100 grid points in each dimension. As The adsorption isotherms model the two-component Langmuir model with $\ln K_1$ =2, $\ln K_2$ = 2, $q_{s,1}$ = 1, and $q_{s,2}$ = 1, were used.

Inspecting Figure S1, we observe that the two-component AED converges (Figures S1d and S1e) only when binary-component solution adsorption data are used in the AED calculations (Figures S1a and S1b). In contrast, when only single-component adsorption isotherm data are used (Figure S1c), we obtain a good estimation of K_1, but K_2 does not converge.

From a mathematical perspective, this can be explained by the solution of eq S4 for component 1, which by using the kernel function from eq S5 depends both on C_1 and C_2. Without competition data,

eq S4 for component 1 becomes zero for all data points in the single-component adsorption data for component 2. In other words, adsorption isotherm for binary-solute solutions of components 1 and 2 are needed to converge to both energy sites, as seen in Figures S1d and S1e.

The exercise above showed that we need binary-component adsorption data to get a convergence. However, the required C2:C1-ratios for conducting two-component AED calculations have not been thoroughly investigated. Results in Figure 3, which used C2:C1-ratios of 1:0, 0:1, and 1:1 (see Figure S3 for experimental data), show successful calculations in the context of a simple adsorption process. Similarly, Figure 4 demonstrates successful calculations using ratios of 1:0, 0:1, 1:3, 1:1, and 3:1 (see Figure S4 for experimental data), where more complex adsorption processes were observed. We can speculate that the required ratios will depend on the adsorption process under study, more complicated processes requiring more data than less complicated processes. Moreover, the quality of the data is important. If the data contains very little noise, we expect that less data are required as compared to more noisy data.

Concentration data used in all calculations

Adsorption isotherm data (synthetic data) were generated using 50 concentration data points for each compound, including 10 single-component data points per component. The mixture concentrations (compound 1: compound 2) were in ratios of 1:1, 1:3, and 3:1 each containing 10 data points. Data were evenly distributed on a natural logarithmic scale from -8 to -0.01. The data are plotted in Figure S2. The experimental data used in this study pertain to two systems.

System 1: Tracer pulse binary adsorption isotherm data for methyl mandelate (MeM) and ethyl mandelate (EtM) on an Eclipse XDB-C8 column were taken from Samuelsson *et al*.[7] The eluent used was 30/70 (v/v) acetonitrile/water. Each adsorption isotherm consists of 36 data points. The mobile phase concentration data are pressented in Figure S3.

System 2: Frontal analysis (FA) and frontal analysis by elution by characteristic points (FACP) binary adsorption isotherm data of benzyl alcohol (BA), 2-phenylethanol (PE), and 2-methyl benzyl alcohol (MBA) on Symmetry C18 column were taken from Quiñones *et al*.[8] The eluent used was 50/50 (v/v) methanol/water. Each adsorption isotherm contained 130 data points for FACP and 50 for FA. The mobile phase concentration data are presented in Figure S4.

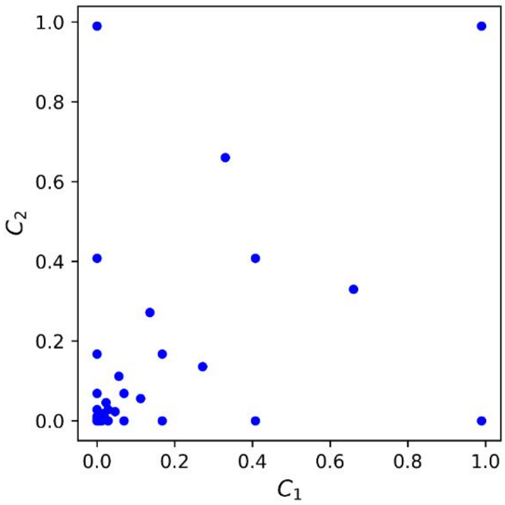

Figure S2: The plot illustrates the relationship between concentration of compound 1 (C_1) and compound 2 (C_2) in the mobile phase. This dataset served as the basis for all synthetic calculations presented in Figures 2 and 3 in the maniscript.

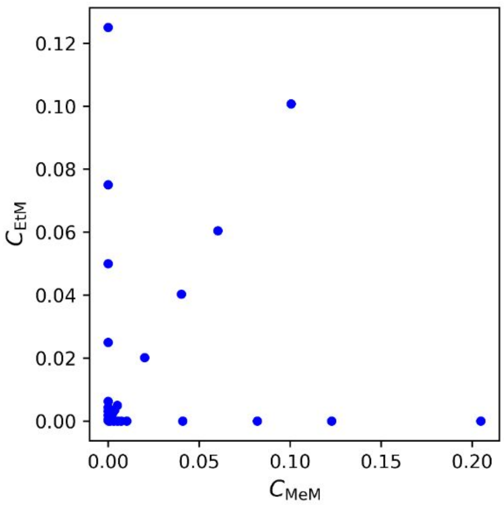

Figure S3: The plot illustrates the relationship between concentration of MeM (C_{MeM}) and EtM (C_{EtM}) in the mobile phase. This dataset served as the basis for all calculations presented in Figure 4.

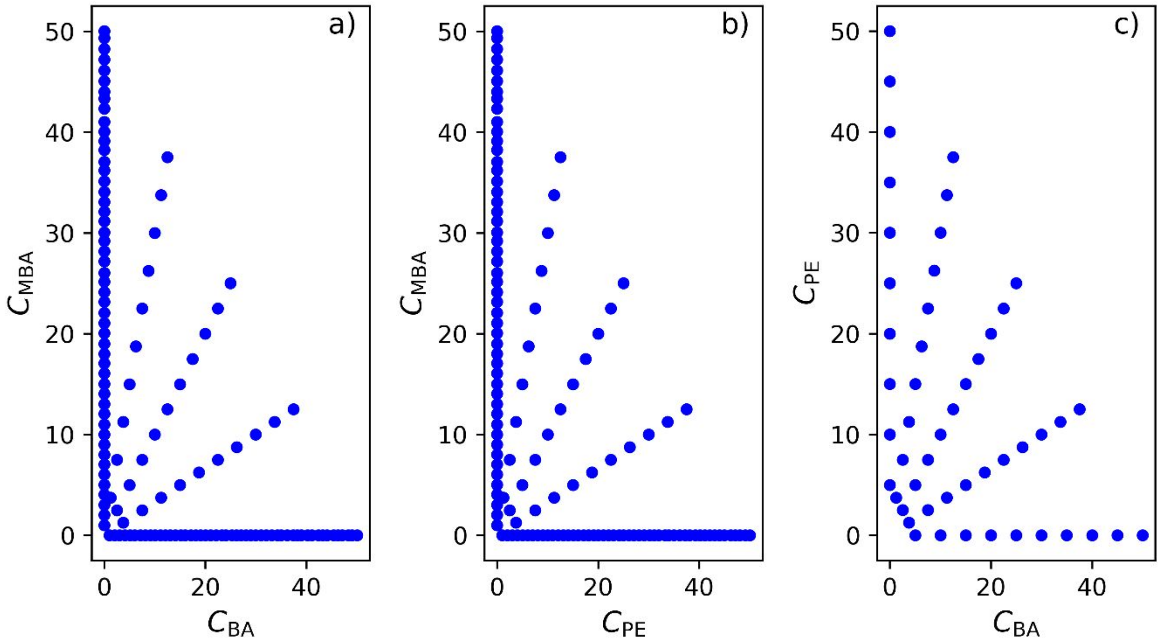

Figure S4: The plot presents the raw mobile phase concentration (C) data used in the two-component AED calculations shown in Figure 5. Subplot a) corresponds to the system BA/MBA, subplot b) to the system PE/MBA, and subplot c) to the system BA/PE.

Model fits

Table S1, present the model fit for the experimental data presented in the main text.

Table S1: Estimated model parameters from non-linear regression (fit) and from the two-component AED. AED 1 is for the first component and AED 2 is for the second compound. All AED calculations were conducted using 100 grid points in each energy space. Number of iterations were 10,000 for MeM/EtM system and 100,000 for the other systems. CPU is the CPU time used in the calculations.

Compound (1/2)		$q_{s,1}$	$q_{s,2}$	K_1	K_2	CPU [s]**
MeM/EtM	Fit	2.75	1.98	0.91	2.31	NA
MeM/EtM	AED 1	2.33	NA	ND	ND	18
MeM/EtM	AED 2	NA	2.23	ND	ND	15
BA/MBA	Fit	123.2	171.7	0.0166	0.0213	NA
BA/MBA	AED 1	174.9	NA	0.0171	0.0242	232
BA/MBA	AED 2	NA	213.8	0.0193	0.0209	253
PE/MBA	Fit	136.4	171.3	0.0241	0.0213	NA
PE/MBA	AED 1	145.0	NA	0.0263	0.0242	286
PE/MBA	AED 2	NA	230.3	0.0221	0.0221	271
BA/PE*	Fit	129.2	147.9	0.0155	0.0216	NA
BA/PE*	AED 1	173.97	NA	0.0137	0.0209	73
BA/PE*	AED 2	NA	151.0	0.0175	0.0226	66

* Here, the frontal analysis experiments were used for single component data.

** All calculations were conducted on a PC running Windows 10 with 16 GB RAM and a 12 Gen Intel Core i5-1235U 1.30 GHz CPU. The calculations for system 2 (BA/MBA, PE/MBA, and BA/PE) took around 260 seconds, except for the BA/PE system which took around 70 seconds, due to lower amount of data points in the adsorption isotherm data, see figure S3. System 1 (MeM/EtM) calculations were significantly faster (below 20 seconds) due to fewer data points (36 points, see figure S2) and fewer iterations (10 times lower) were used in the calculation.

Adsorption isotherm model fit using the two-component AED

Calculating the AED involves solving a minimization problem to identify a function that minimizes the difference between the calculated adsorption isotherm (q_{cal}, see Eqs. S3 and S6) and the experimental adsorption isotherm (q_{exp}).

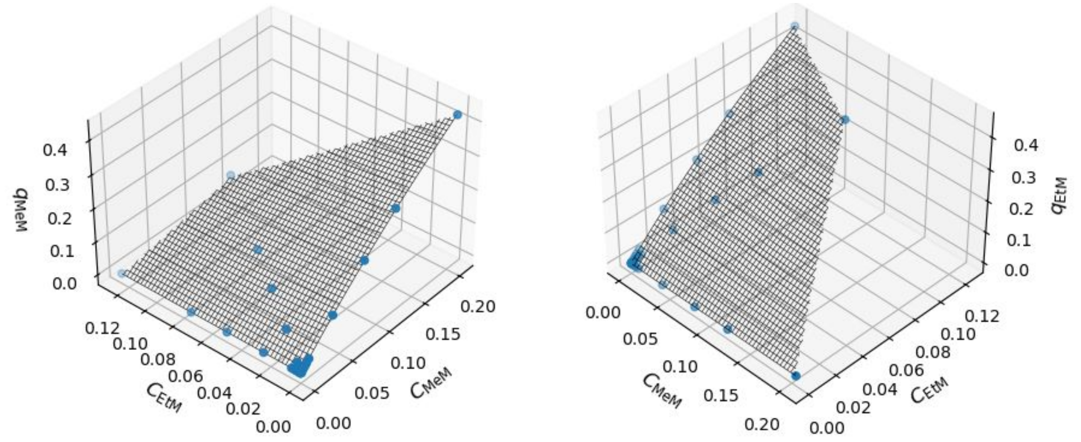

Figure S5: The surface represents the two-component AED adsorption isotherm model fit, and the dots represent the experimental adsorption isotherm data for the MeM/EtM system. Left figure: MeM, and right figure: EtM.

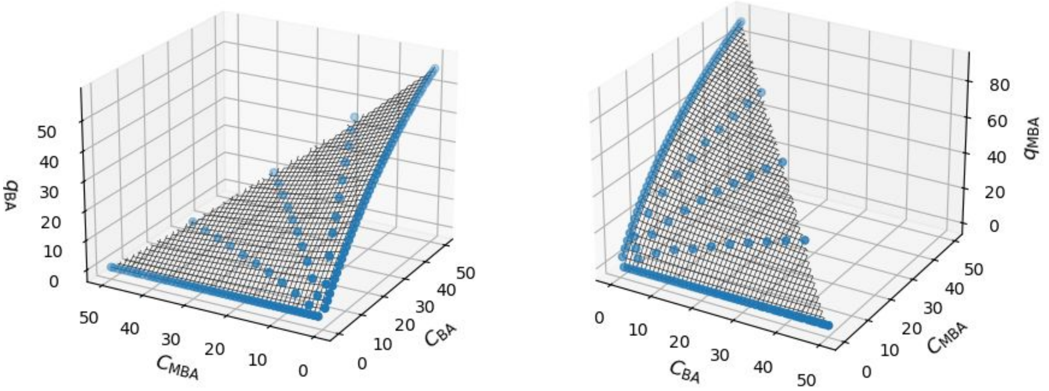

Figure S6: The surface represent the two-component AED adsorption isotherm model fit, and the dots represent the experimental adsorption isotherm data for the BA/MBA. Left figure) for BA and right figure) for MBA.

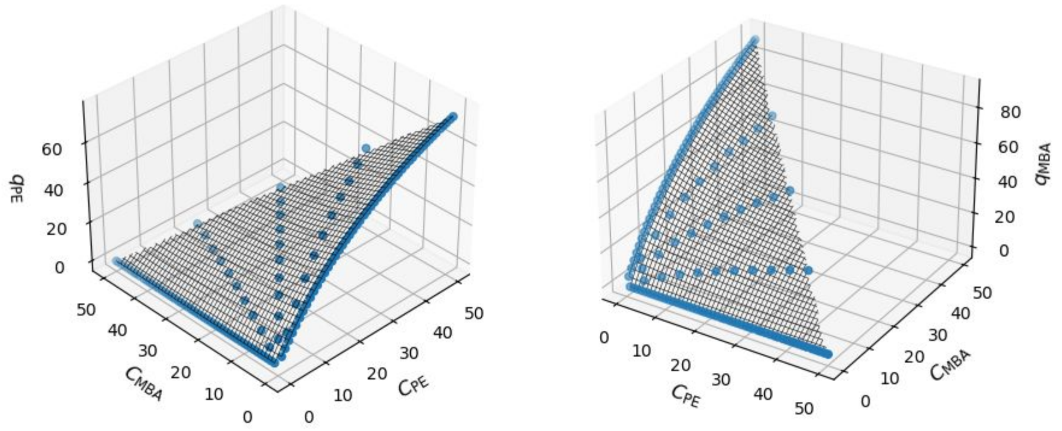

Figure S7: The surface represent the two-component AED adsorption isotherm model fit, and the dots represent the experimental adsorption isotherm data for the PE/MBA. Left figure) for PE and right figure) for MBA.

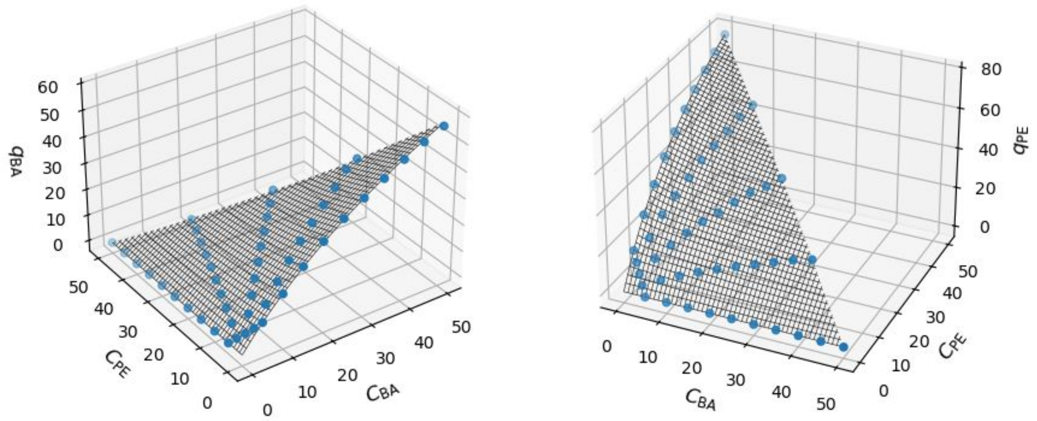

Figure S8: The surface represent the two-component AED adsorption isotherm model fit, and the dots represent the experimental adsorption isotherm data for the BA/PE. Left figure) for BA and right figure) for PE.

In essence, eqs S1 and S4 provide a unique approach to fitting adsorption isotherm models without assuming heterogeneity. Instead, it assumes that the local adsorption isotherm (the kernel function) is valid within a very small energy space. This contrasts with classical model fitting, where the adsorption isotherm model is assumed to be valid across the entire concentration range. Figure S5 illustrates the comparison between calculated and experimental adsorption isotherms for the MeM/EtM system. Similarly, Figure S6 presents the results for the BA/MBA system, Figure S7 for the PE/MBA system, and Figure S8 for the BA/PE system.

References

(1) Guiochon, G.; Shirazi, D. G.; Felinger, A.; Katti, A. M. *Fundamentals of Preparative and Nonlinear Chromatography*, 2nd ed.; Academic Press: Boston, MA, 2006.

(2) Stanley, B. J.; Guiochon, G. Numerical Estimation of Adsorption Energy Distributions from Adsorption Isotherm Data with the Expectation-Maximization Method. *J. Phys. Chem.* **1993**, *97* (30), 8098–8104. https://doi.org/10.1021/j100132a046.

(3) Samuelsson, J.; Franz, A.; Stanley, B. J.; Fornstedt, T. Thermodynamic Characterization of Separations on Alkaline-Stable Silica-Based C18 Columns: Why Basic Solutes May Have Better Capacity and Peak Performance at Higher pH. *J. Chromatogr. A* **2007**, *1163* (1–2), 177–189. https://doi.org/10.1016/j.chroma.2007.06.026.

(4) Stanley, B. J.; Krance, J. Analysis of Active Sites and Heterogeneity in Commercial Reversed-Phase Octadecylsilanated Silica with Numerically Calculated Sorption Distributions. *J. Chromatogr. A* **2003**, *1011* (1–2), 11–22.

(5) Waluga, T.; Skiborowski, M. Using Adsorption Energy Distribution for Parameter Estimation of Competitive Cofactor Coupled Enzyme Reaction. *Processes* **2023**, *11* (9), 2686. https://doi.org/10.3390/pr11092686.

(6) Stanley, B. J.; Bialkowski, S. E.; Marshall, D. B. Analysis of First-Order Rate Constant Spectra with Regularized Least-Squares and Expectation Maximization. 1. Theory and Numerical Characterization. *Anal. Chem.* **1993**, *65* (3), 259–267. https://doi.org/10.1021/ac00051a013.

(7) Samuelsson, J.; Arnell, R.; Diesen, J. S.; Tibbelin, J.; Paptchikhine, A.; Fornstedt, T.; Sjoberg, P. J. R. Development of the Tracer-Pulse Method for Adsorption Studies of Analyte Mixtures in Liquid Chromatography Utilizing Mass Spectrometric Detection. *Anal. Chem.* **2008**, *80* (6), 2105–2112. https://doi.org/10.1021/ac702399a.

(8) Quiñones, I.; Ford, J. C.; Guiochon, G. High-Concentration Band Profiles and System Peaks for a Ternary Solute System. *Analytical Chemistry* **2000**, *72* (7), 1495–1502. https://doi.org/10.1021/ac9909406.
